# Physiological constraints dictate toxin spatial heterogeneity in snake venom glands

**DOI:** 10.1186/s12915-022-01350-y

**Published:** 2022-06-27

**Authors:** Taline D. Kazandjian, Brett R. Hamilton, Samuel D. Robinson, Steven R. Hall, Keirah E. Bartlett, Paul Rowley, Mark C. Wilkinson, Nicholas R. Casewell, Eivind A. B. Undheim

**Affiliations:** 1 Centre for Snakebite Research & Interventions, Liverpool School of Tropical Medicine, Pembroke Place, Liverpool, L3 5QA UK; 2 Centre for Advanced Imaging, University of Queensland, St Lucia, QLD 4072 Australia; 3 Centre for Microscopy and Microanalysis, University of Queensland, St Lucia, QLD 4072 Australia; 4 Institute for Molecular Bioscience, University of Queensland, St Lucia, QLD 4072 Australia; 5 Centre for Ecological and Evolutionary Synthesis, Department of Biosciences, University of Oslo, PO Box 1066 Blindern, 0316 Oslo, Norway

**Keywords:** Venom, Snake, Behaviour, Adaptation, Evolution, Mass spectrometry imaging

## Abstract

**Background:**

Venoms are ecological innovations that have evolved numerous times, on each occasion accompanied by the co-evolution of specialised morphological and behavioural characters for venom production and delivery. The close evolutionary interdependence between these characters is exemplified by animals that control the composition of their secreted venom. This ability depends in part on the production of different toxins in different locations of the venom gland, which was recently documented in venomous snakes. Here, we test the hypothesis that the distinct spatial distributions of toxins in snake venom glands are an adaptation that enables the secretion of venoms with distinct ecological functions.

**Results:**

We show that the main defensive and predatory peptide toxins are produced in distinct regions of the venom glands of the black-necked spitting cobra (*Naja nigricollis*), but these distributions likely reflect developmental effects. Indeed, we detected no significant differences in venom collected via defensive ‘spitting’ or predatory ‘biting’ events from the same specimens representing multiple lineages of spitting cobra. We also found the same spatial distribution of toxins in a non-spitting cobra and show that heterogeneous toxin distribution is a feature shared with a viper with primarily predatory venom.

**Conclusions:**

Our findings suggest that heterogeneous distributions of toxins are not an adaptation to controlling venom composition in snakes. Instead, it likely reflects physiological constraints on toxin production by the venom glands, opening avenues for future research on the mechanisms of functional differentiation of populations of protein-secreting cells within adaptive contexts.

**Supplementary Information:**

The online version contains supplementary material available at 10.1186/s12915-022-01350-y.

## Background

Venoms are among the most frequently evolved adaptive traits in animals and have emerged on over 100 occasions throughout the animal kingdom [1]. On many of these occasions, proteins with physiological roles have been functionally co-opted as toxins, and this process is often followed by large gene family expansions and structural and functional diversification [2]. The evolution of these biochemical arsenals was also accompanied by the emergence of associated venom-producing tissues, venom-delivering structures, and specialised behaviours associated with venom use. Together, these traits form *venom systems*, which are integrated systems with phenotypes that can be measured as clearly definable sets of pharmacological properties.

As integrated structures of measurable traits, venom systems are amenable models for studying how traits facilitate or constrain the evolution of other traits across different levels of biological complexity [1]. For example, the cone snails *Conus geographus* and *Conus marmoreus* can behaviourally control the composition of injected venom to contain toxins predominantly involved in either predation or defence [3], with the functionally distinct toxins evolving under different types of selection [3, 4]. These specialised behaviours are enabled by spatial segregation of toxins across the venom gland, and there is thus a close link between the functional morphology of venom-producing tissues and the evolution of toxins and associated behaviours.

Spatially distinct distributions of toxins across the venom glands are not just found in some cone snails but have also been documented in other venomous lineages such as sea anemones [5–8], assassin bugs [9, 10], elapid snakes [11, 12], and centipedes [13, 14]. However, relationships between toxin distributions are not always clear. In sea anemones and assassin bugs, toxin function is closely related to their distribution across the sea anemone’s functional anatomy or assassin bug venom gland compartments [5–10]. In contrast, distinct toxin distributions in centipedes are thought to primarily reflect biochemical constraints on toxin production in secretory cells [13]. Such heterogeneous populations of secretory cells have also been found in the venom glands of cone snails [15, 16], as well as other protein-secreting tissues [17], raising the question whether the apparent link between toxin distribution and function is an adaptation to toxin use or a phenomenon that reflects more fundamental physiological limitations of protein-secreting tissues.

A challenge with studying the ecological functions of venom is collecting venom secreted naturally in a functionally relevant context. While the oral venom systems of snakes evolved to facilitate prey capture via biting, spitting cobras also exhibit venom-spitting behaviour, which represents an exclusively defensive use of venom. Defensive venom spitting is underpinned by behavioural and morphological adaptations [18–21] and evolved on three separate occasions in cobras (*Naja* spp.) and their near relatives [22–24]. Interestingly, each spitting lineage has also upregulated venom phospholipase A_2_ (PLA_2_) toxins, which work synergistically with cobra cytotoxins (cytotoxic three-finger toxins) to elicit strong responses in sensory neurons, and hence, venom spitting targeting the eyes of an aggressor likely confers a degree of protection through inducing pain [22]. This adaptation does not preclude the predatory use of venom via biting, yet such algogenic (pain-inducing) properties could be disadvantageous to prey capture because they likely exacerbate flight responses, thereby creating a potential evolutionary conflict between the defensive and predatory properties of the venom. This conflict would be avoided by being able to control the composition of secreted venom, in a similar manner to that previously described for certain invertebrates, such as the aforementioned species of cone snails [3]. However, such venom partitioning has yet to be described in vertebrates. Spitting cobras represent a model where one might expect the strongest selection towards this complex functional morphological and behavioural adaptation.

Here, we test the hypothesis that the differential distribution of toxins in snakes represents the physiological adaptations that allow the secretion of functionally distinct venoms and thereby reduce the evolutionary conflict between defensive and predatory venom components. However, using a combination of mass spectrometry imaging and venom compositional and functional analyses, we instead find that the distributions of functionally distinct toxins across snake venom glands likely reflect spatial correlation of closely related gene products and that toxin heterogeneity is a seemingly universal feature of the venom glands of front-fanged snakes. We propose that distinct toxin distributions across the venom glands are the result of physiological constraints on secretory tissues—such as the presence of different populations of secretory cells that have an adapted molecular machinery for toxin biosynthesis and secretion—rather than the result of adaptation and that the spatial toxin expression patterns that arise from these constraints may in some cases serve as exaptations that allow venomous organisms to circumvent evolutionary conflicts between toxins of different function.

## Results

### Toxin distributions across the venom gland reflect both function and relatedness

**Fig. 1:**
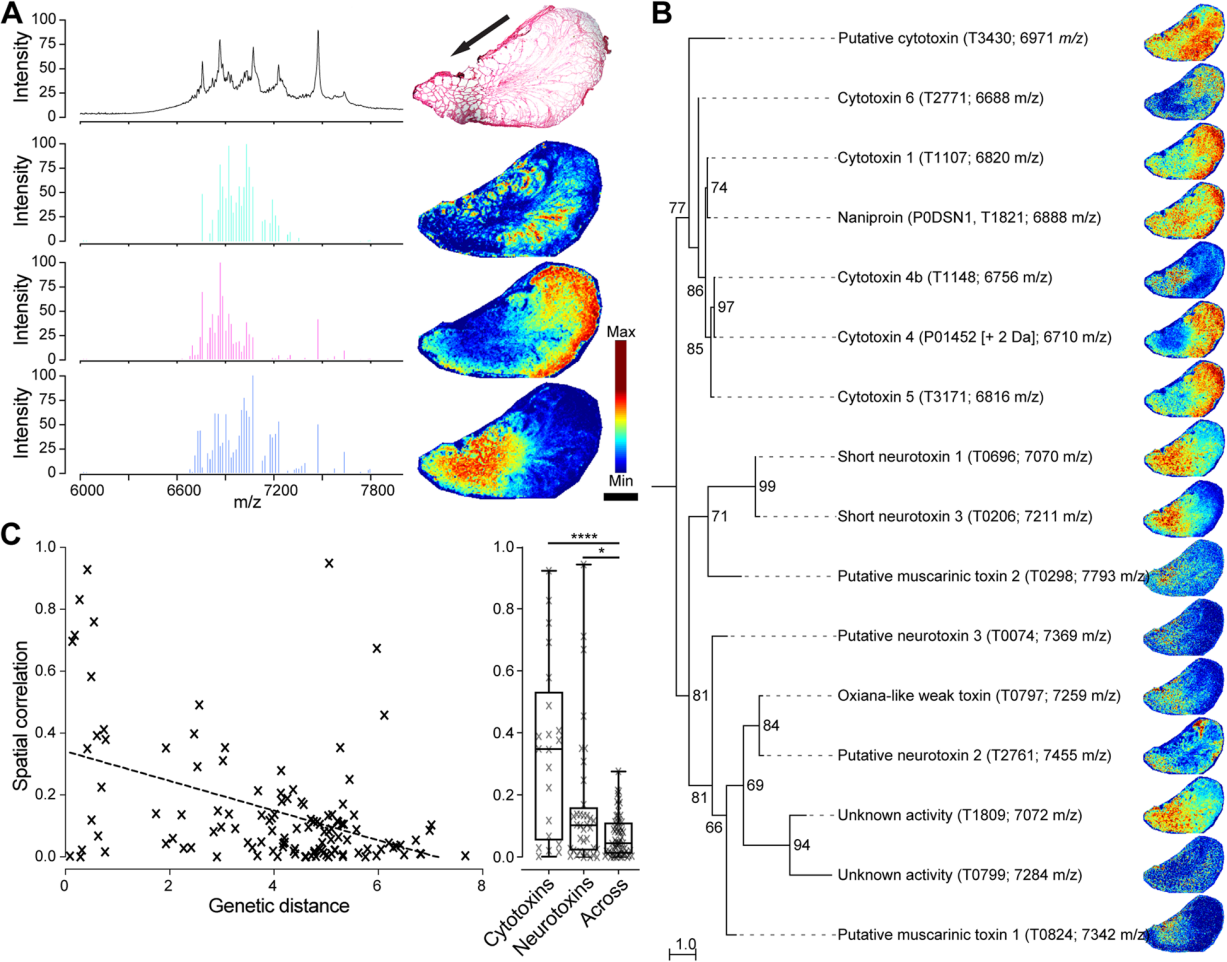
The venom gland of *N. nigricollis* contains functionally distinct clusters of related toxins. **A** Detected toxins can be grouped into three clusters with distinct distributions across the venom gland of *N. nigricollis.* The top spectrum shows the normalised across-tissue averaged spectrum in the *m/z* region corresponding to 3FTx—the dominant components of *N. nigricollis* venom (full spectrum in Additional file 1: Fig. S1). The below centroid spectra are extracted from each group clustered by probabilistic latent semantic analysis (pLSA), where distributions across the gland are displayed as contrast-optimised heatmaps on the right. The top image shows the section stained with haematoxylin and eosin (H&E) post-MSI acquisition, and the direction of venom secretion is indicated by the arrow. **B** Toxin distributions are correlated with functional and phylogenetic relationships, as shown by comparing the phylogenetic relationship and distributions of identified 3FTx. The displayed 3FTx phylogeny was reconstructed by maximum likelihood (ML) under the FLU+G4 model (left, bootstrap support at nodes, displayed as mid-point rooted), while their distribution across the venom gland as determined by MSI is shown as contrast-optimised heatmaps on the right. **C** The relationship between function, relatedness, and distribution is further supported by a significant correlation between pairwise ML distances and spatial correlations of the identified 3FTx (left; Spearman rank: *P* < 0.001, *r* = − 0.337, 95% CI = − 0.491 to − 0.163, *R*^2^ = 0.1944) and significantly higher spatial correlations among toxins within compared to between each functional class (right; Mann-Whitney two-tailed: *P* = 0.0481 and < 0.0001 for neurotoxins and cytotoxins, respectively). Heatmap legend is shown in **A**, and scale bar represents 2 mm

Spatially distinct distributions of toxins have previously been observed in the venom glands of the non-spitting cobra *Naja subfulva* [11]. To investigate whether the venom glands from the related spitting cobra species *Naja nigricollis* also exhibit discrete spatial toxin distributions, we applied matrix-assisted laser desorption ionisation (MALDI) mass spectrometry imaging (MSI). The resulting MSI spectra were dominated by strong signals in the region of mass-to-charge ratio (*m*/*z*) corresponding to masses typical of three-finger toxins (3FTx), most of which were confined to distinct regions of the venom gland (Fig. 1, Additional file 1: Fig. S1, Table S1). This multi-locus gene family, which encodes both neurotoxic and cytotoxic 3FTx toxin isoforms, has previously been demonstrated to be the most abundant protein family detected in the venom of cobras [22, 25]. Cluster analysis of the corresponding spectra revealed three distinct groups distributed mainly in the anterior or posterior parts of the venom gland (Fig. 1A), suggesting that the components in the venom gland of *N. nigricollis* form at least two spatially distinct clusters.

The concerted action of cytotoxic 3FTxs and PLA_2_s in spitting cobra venom is thought to be responsible for stimulating algogenic effects via activation of sensory neurons in the targeted animal following defensive spitting [22]. To examine whether the observed toxin clusters present in the *N. nigricollis* venom gland could represent adaptations to defensive venom spitting, we matched MSI peaks to known toxin masses and then mapped their distribution and annotated functional activity onto their molecular phylogeny (Fig. 1B). This analysis revealed that toxins form functionally distinct (almost exclusively so) clusters, and specifically, that cytotoxic and neurotoxic 3FTx form distinct clades, suggesting that their distribution may also be explained by relatedness. Indeed, while we detected a weak but significant relationship between the spatial correlation of toxins and their genetic distance, 3FTx were also significantly spatially correlated with toxins belonging to the same functional class compared to those of a different functional class (Fig. 1C).

### Spitting cobras do not actively modulate their venom composition

While the discrete distributions of toxin types observed in the venom gland of *N. nigricollis* could be an adaptation to functionally distinct venoms (i.e. posterior algogenic cytotoxins vs anterior ‘neurotoxins’), they can also be explained by a combination of developmental (e.g. stem cell proliferation) and biochemical (e.g. protein folding) constraints that favour regional specialisation of toxin production of structurally similar paralogues. To explore this further, we collected venom from three spitting cobra species representing multiple elapid snake lineages that have independently evolved defensive venom spitting (*N. nigricollis*, *N. pallida*, and the cobra relative, *Hemachatus haemachatus*). For each species, venom was collected from both spitting events (i.e. via manually stimulated venom spitting, referred to as ‘spat’ from here on; see Additional file 1: Fig. S2) and biting events (i.e. via typical venom extraction, referred to as ‘milked’ from here on), with milked venom samples extracted following the collection of venom spits.

**Fig. 2:**
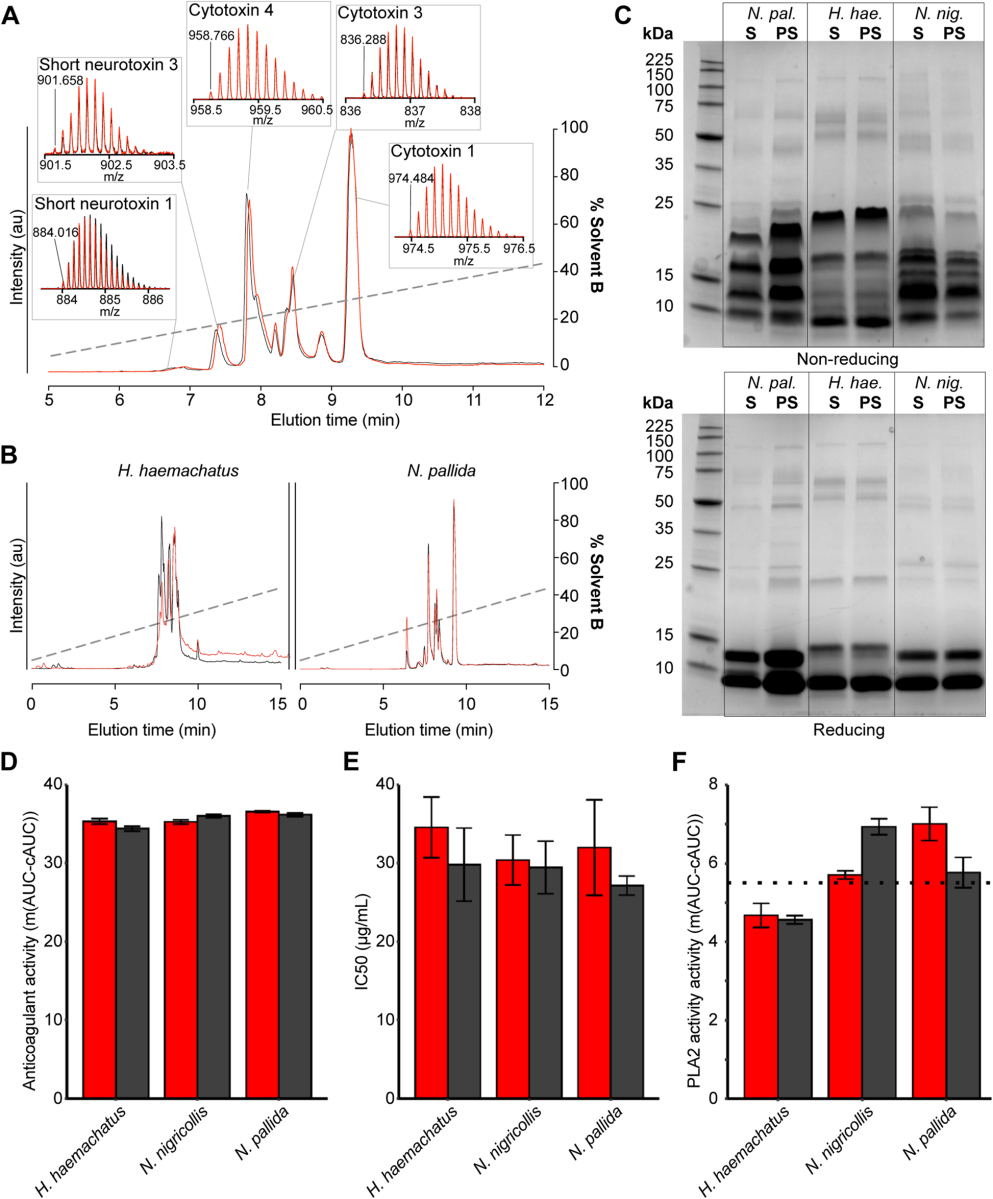
The composition and activity of venoms spat by and milked from spitting cobras are nearly identical. **A** Overlaid total ion chromatograms (TICs) and isotope distributions of key toxins from spat (red) and milked (black) venoms with different distributions in the venom gland from *N. nigricollis*, showing that their peptide toxin composition is identical. **B** Comparisons of spat and milked venom total ion counts (TICs) from representatives of two independently evolved clades of ‘spitting cobras’ show that they are either identical or have only minor differences in the abundance of peptide toxins. **C** Non-reduced and reduced SDS-PAGE of spat (S) or post-spit milked (PS) venom suggest that the composition of high-molecular-weight protein toxins is also identical or highly similar. *Abbreviations*: *N. pallida* (*N. pal.*), *H. haemachatus* (*H. hae*.), *N. nigricollis* (*N. nig.*). **D** Anticoagulant activity of spat and milked venom on citrated bovine plasma, measured as the sample area under the curve (AUC) minus the mean (*m*) control AUC, averaged (*m* (AUC − cAUC)), across four replicates (see Additional file 3). **E** Cytotoxic activity of spat and milked venom measured via MTT cell viability assay and displayed as the venom concentration that resulted in a 50% reduction in cell viability (IC_50_) across three replicates (see Additional file 4). **F** Enzymatic PLA_2_ activity of spat and milked venom, measured kinetically and displayed as *m*(AUC − cAUC), across three replicates (see Additional file 5). Error bars represent the standard error of the mean (SEM) of triplicate readings. Dotted lines in **F** indicate the activity of positive control venom (*Daboia russelii*) selected for its high PLA_2_ activity to contextualise the findings shown here

Quantification of the resulting venom yields demonstrated, as anticipated, that defensive spitting results in the ejection of substantially less venom than a biting event, with the mean weight of venom spits ranging from 1.8 to 8.3 mg (wet weight; 0.9–4.9 mg dry weight) compared with 529.4–958.2 mg (wet weight; 118.3–309.8 mg dry weight) as a result of manual venom extractions used here as a proxy for expulsion via biting (Additional file 1: Fig. S3). Compositional profiling of the resulting venoms revealed highly similar venom compositions between spat and milked samples, irrespective of the sampled species (Fig. 2 and Additional file 1: Fig. S4). Specifically, the use of both cation exchange and reversed-phase high-performance liquid chromatography (RP-HPLC) resulted in near-identical venom profiles, with no evidence of differentially produced venom proteins, and only slight differences observed in the relative abundances of a small number of protein peaks found between the spat and milked samples, which were most evident in the venom of *H. haemachatus* (Fig. 2 and Additional file 1: Fig. S4). The use of sodium dodecyl-sulphate polyacrylamide gel electrophoresis (SDS-PAGE) provided consistent and complementary data, with near-identical protein profiles and band intensities observed between the spat and milked samples (Fig. 2). These findings demonstrate that, despite differential toxin localisation observed within the venom gland, spitting cobras exhibit consistent venom toxin composition irrespective of the mode of venom stimulation, thus likely to not eject bipartite venoms depending on the ecological scenario (i.e. predatory vs defensive venom use) they are presented with.

To exclude the possibility that subtle differences in certain toxin abundances might influence the overarching functional phenotype of the venoms, we subjected the spat and milked venom samples to a panel of in vitro functional analyses. Specifically, we quantified the coagulopathic (Additional file 1: Fig. S5), cytotoxic (Additional file 1: Fig. S6), phospholipase (Additional file 1: Fig. S7), and metalloproteinase (Additional file 1: Fig. S8) activities of the venom samples using previously defined plasma-based absorbance [26], methyl tetrazolium (MTT)-based cell viability [27], pH-based colourimetric [28], and substrate-specific fluorescent assays [29], respectively. Consistent with the known venom compositions of the three species under study [22, 25], each venom exhibited potent and comparable anticoagulant, phospholipase, and cytotoxic activities (Fig. 2D–F), though little metalloproteinase activity was observed (Additional file 1: Fig. S8). Crucially, and in line with the venom-compositional profiles, no statistically significant differences were detected between the spat and milked venoms in any of the functional bioassays (two-way analysis of variance (ANOVA): coagulation, *F* = 0.82, df = 1, *P* = 0.38; cytotoxicity, *F* = 1.96, df = 1, *P* = 0.18; phospholipase, *F* = 0.03, df = 1, *P* = 0.86; metalloproteinase, *F* = 0.41, df = 1, *P* = 0.53). These findings further demonstrate that spitting cobras do not exhibit bipartite venoms, and the combination of toxin compositional and functional data strongly suggests that the distinct toxin distributions detected in the venom gland are not the result of adaptation related to the evolution of defensive venom spitting.

### Physiological constraints underlie differential toxin production in snake venom glands

**Fig. 3:**
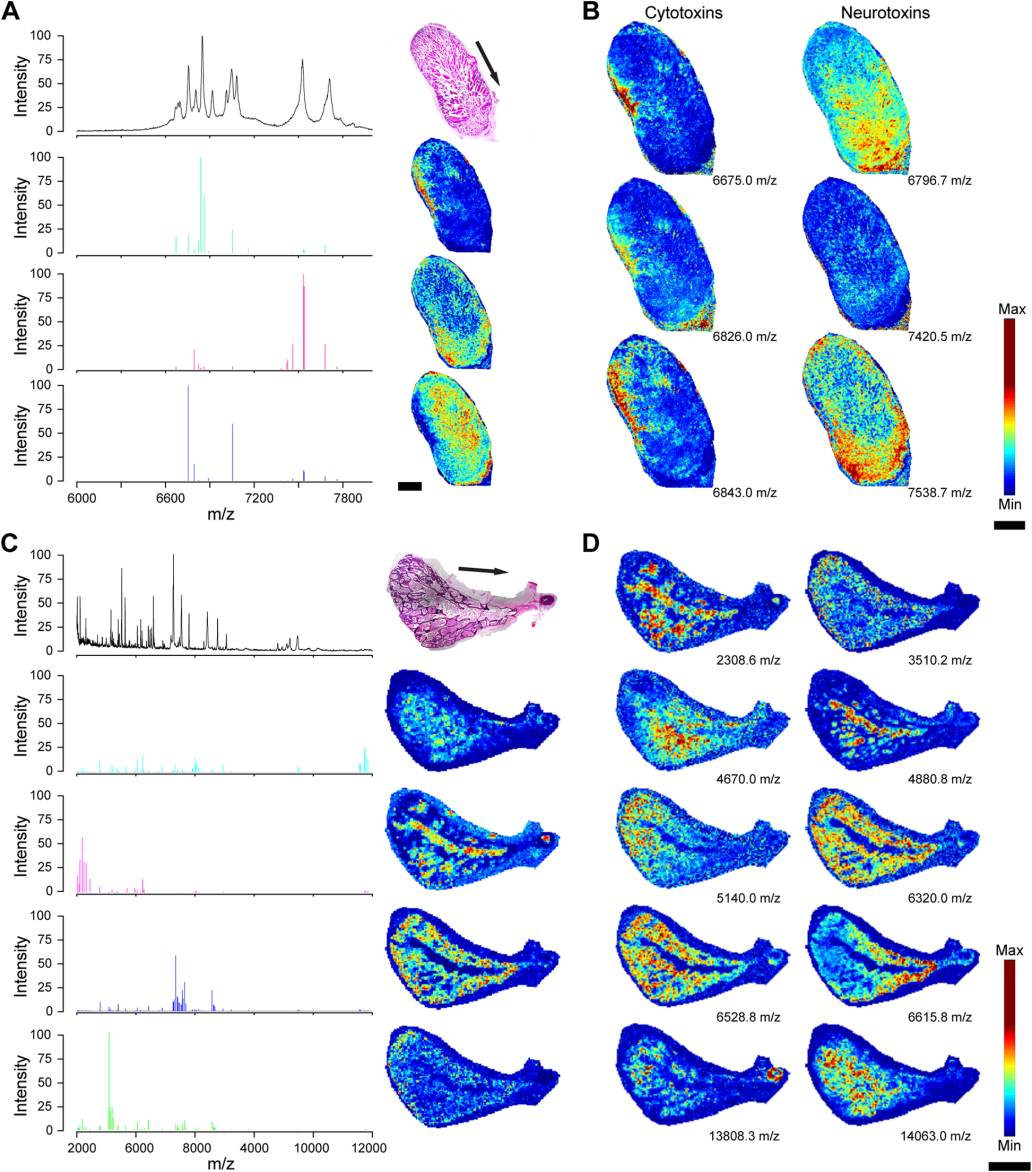
Distinct toxin distributions across the venom glands are taxonomically widespread in snakes. **A** pLSA analysis of MSI spectra from the venom gland of *N. subfulva*. The top spectrum shows the normalised across-tissue averaged spectrum in the *m*/*z* region corresponding to 3FTx—the dominant components of *N. subfulva* venom. The below centroid spectra are extracted from each group clustered by pLSA, where distributions across the gland are displayed as contrast-optimised heatmaps on the right. The H&E-stained section is shown at the top with an arrow indicating the direction of venom secretion. **B** Distributions of peaks corresponding to toxins with known activities are shown as contrast-optimised heatmaps. Accession numbers for cytotoxins are UniProt P01448, P01473, and P01474 and for neurotoxins, UniProt P01424, P01400, and GenBank GIJM01004310.1. **C** pLSA analysis of MSI spectra from the venom gland of *C. rhodostoma*. The top spectrum shows the normalised across-tissue averaged spectrum across the full acquired *m*/*z* range. The below centroid spectra are extracted from each group clustered by pLSA, where distributions across the gland are displayed as contrast-optimised heatmaps on the right. The H&E-stained section is shown at the top with an arrow indicating the direction of venom secretion. **D** Distributions of major peaks corresponding to the *m*/*z* values given below each image are shown as contrast-optimised heatmaps

Given the unlikely adaptive value of the observed toxin distributions to defensive venom spitting, we next examined whether similar patterns of heterogeneity are present in the venom glands of related non-spitting cobras. MSI of the venom glands from *N. subfulva* showed the same spatial distribution of toxins as *N. nigricollis*, with cytotoxins and neurotoxins located mainly distally and proximally to the fang, respectively (Fig. 3A, B). Although cytotoxins are the toxins thought to stimulate algogenic responses fundamental for defensive venom spitting, their action is significantly potentiated by PLA_2_ toxins [22]. However, while cytotoxins are largely confined to the posterior half of the venom gland, venom PLA_2_s are found distributed throughout the venom glands of both *N. nigricollis* (Additional file 1: Fig. S9) and *N. subfulva* [11]. These differences in the distributions between cytotoxins and PLA_2_ suggest there are no co-developmental associations between the molecular defensive innovations of cobras, and that their distributions are probably the result of other, non-adaptive factors.

To determine whether heterogeneous toxin distributions could instead represent a more widespread phenomenon reflecting fundamental properties of the snake venom glands, we imaged the venom glands of the distantly related viperid snake *Calloselasma rhodostoma*. This species lacks defence-specific behaviours akin to spitting and is thought to use its venom mainly for predatory purposes. Although a clustering analysis of MSI spectra did not identify distinct regions along the length of the venom glands (Fig. 3C), we observed distinct distributions for prominent peaks that were largely restricted to either posterior or anterior ends of the glands (Fig. 3D). These putative venom protein and peptide peaks also included those with masses that closely matched those of known *C. rhodostoma* venom PLA_2_ (13,808 and 14,063 *m*/*z*). Taken together, these results suggest spatially heterogeneous toxin localisation is a common feature of snake venom glands and is likely not associated with functional adaptations to venom-associated behaviours or toxin functions. Instead, heterogeneous toxin distributions across snake venom glands likely reflect fundamental physiological constraints on toxin production stemming from, for example, limitations on the molecular machinery for toxin biosynthesis and secretion.

## Discussion

The ability to control the composition of secreted venom is known from three venomous lineages: cone snails, assassin bugs, and scorpions. Of these, cone snails and assassin bugs possess the greatest control, with some species having been shown to selectively secrete different venom components [3, 9, 10], presumably through neuronal innervation of different regions of their venom glands. This direct mode of venom modulation contrasts with that of certain scorpions, which are thought to indirectly control the composition of their venoms by regulating the *amount* of venom secreted—the initial venom secretions are rich in K^+^ ions, while consecutive droplets contain the peptidic venom components [30]. Indirect venom modulation is thought to be enabled by storing functionally distinct toxins along the length of the venom gland [1], which is a phenomenon also found in snake venom glands. We therefore hypothesised that snakes were likely to be able to indirectly control the composition of their secreted venom. However, our results demonstrate that this is not the case and that the distinct toxin distributions observed here in snake venom glands are not adaptations to modulating venom.

Cytotoxic 3FTxs are seemingly a cobra-specific defensive innovation that have evolved in association with hooding in cobras [23]. It is therefore possible that the restriction of cytotoxic 3FTXs to the posterior regions of the venom gland observed here could represent an adaptation that enables mainly neurotoxic venom to be secreted during predatory bites. However, although spitting results from much smaller venom gland-associated muscle contractions than bites [31], the yields of spat venom collected here were sufficient to expel the full toxin cocktail, reducing the likelihood of the above explanation. This initially appears to be in contradiction with previous evidence from *Naja pallida*, in which a 9-kDa toxin (likely a three-finger toxin) observed in the first spit was absent after 20 spitting events [32]. These findings perhaps suggest that storing defensive components in the posterior region of the gland may serve to retain these for defensive purposes. However, here, we were unable to induce any snake to spit more than eight consecutive times—a number that is consistent with previous studies on cobra spitting behaviour [33]. During such instances, which appear to represent an ecologically relevant number of spits rather than artificially induced depletion of the venom gland, we find that spat (defensive) venom is both compositionally and functionally identical to venom milked via biting (a proxy for an offensive bite) for multiple spitting species of snake.

Although posterior glandular storage of cytotoxins appears at first glance to be counterproductive, as ‘defensive’ cytotoxins would seemingly be redundant during the use of venom for prey capture, it is worth noting that these toxin constituents are among the most abundant of all those found in cobra venoms [22, 25]. Indeed, while cytotoxins are implicated in local cytotoxicity following defensive human snakebites by spitting cobras [23, 34, 35] and for stimulating pain (in conjunction with potentiating PLA_2_ toxins) during defensive spitting [22], these toxins may also play a role in prey capture by acting as ‘spreading factors’ by facilitating local cellular disruption that in turn aids the distribution of neurotoxic components to neuromuscular junctions where they cause paralysis [36, 37]. Thus, cytotoxins may be of considerable functional importance even in species that prioritise neurotoxicity as the means of prey capture. Furthermore, the distribution pattern of cytotoxins and neurotoxins appears to be a common feature across cobras, irrespective of their ability to spit venom, and is therefore not likely to be preadaptation to the evolution of this trait. Instead, this defence-specific behaviour appears to have evolved despite the location of defensive toxins at the posterior end of the gland: this organisation precludes the ability to selectively secrete such toxins by indirect venom modulation—i.e. through regulating the amount of spat venom [1], thereby increasing the metabolic cost and potential evolutionary conflict associated with spitting behaviour.

## Conclusions

Overall, our data suggest that the spatial heterogeneity of toxins observed in the spitting cobra (*N. nigricollis*) venom gland is not the result of adaptation associated with the evolution of the defensive spitting trait. Indeed, comparable distributions observed in the venom glands of the non-spitting cobra (*N. subfulva*), and evidence of spatial heterogeneity in that of a representative, distantly related, viper (*C. rhodostoma*), suggest instead that differential toxin localisations may be a common feature in the snake venom gland. These data seem likely to reflect molecular and physiological constraints on toxin production within the gland and hint at differential production and/or storage of functionally and genetically related toxins in disparate physical locations. Although the biological basis for these differential localisations remains unclear, our data highlight several future research avenues to better understand toxin production, storage, and protection against autotoxicity in these specialised venom secretory tissues. The further application of mass spectrometry imaging, coupled with complimentary expression profiling of different cell populations via single-cell sequencing, might be particularly revealing in this regard, since recent research has suggested that different venom gland cell populations might differentially contribute toxins to the overall venom cocktail [12]. These findings also suggest that protein neofunctionalisation, as observed here in the case of 3FTx cytotoxins, may constrain the localisation of new proteins to specific regions of secretory tissues and thus highlights venom glands as exciting models for studying the mechanisms underlying the heterogeneity of secretory cells within defined functional contexts.

## Methods

### Venom gland imaging

The venom glands were dissected from a single individual of *N. nigricollis* (Tanzania) and *C. rhodostoma* (captive bred) following euthanasia via an overdose of injectable pentobarbital sodium (PentoJect®). Following dissection, one venom gland from each individual was processed using the same protocol as previously described for *N. subfulva* [11], which removes lipids as well as most non-fixed, non-peptidic analytes and thereby improves signals from peptidic toxins. Briefly, the venom glands were fixed in RCL2 (Alphelys, France), dehydrated through 70%, 90%, and 100% ethanol (vol/vol), cleared with xylene, and embedded in paraffin. The sections were cut at 7 μm thickness from cold paraffin block using a microtome, placed directly onto an indium-tin oxide (ITO) glass slide, attached to the section by heating to 57 °C, and allowed to cool before the paraffin was removed using xylene. We then used an ImagePrep vibrational vaporisation-deposition system (Bruker) to apply the matrix onto the slide (105 mg α-cyano-4-hydroxycinnamic acid (CHCA), 8 mL acetonitrile, 7 mL water, 30 μL trifluoroacetic acid) using the standard Bruker ImagePrep CHCA application method. MSI data were acquired using Flex Imaging 4.1 and Flex Control 3.4 to operate a MALDI-TOF/TOF (Bruker Ultraflex III, Bruker, Bremen, Germany) in linear positive mode. Scans were acquired over a mass range of *m*/*z* 1000–15,000 at 200 Hz, 50 μm spatial resolution, 600 shots, and using a medium laser size.

Data was analysed post-acquisition using Flex Imaging 4.1 and SCILs Lab MVS premium (Bruker Daltonics). To examine whether analytes (including both toxins and non-toxins) formed groups with distinct distributions, we used pLSA with random initiation and reconstructed mass spectra for each component from the loadings plot data. In order to avoid over-estimating the number of potentially distinct regions, we used the minimal number of categories for the pLSA that identified the off-tissue region as a separate group (four groups; the matrix group is not shown in Fig. 1). The MSI data was baseline subtracted using the convolution algorithm and are displayed with weak denoising.

To look for the distributions of specific toxins in *N. nigricollis*, observed peaks were compared to the average oxidised masses of all toxins known from the venom of *N. nigricollis*, which were obtained from UniProtKB [38] and the recently published *N. nigricollis* venom gland transcriptome (GenBank TSA GIJF00000000.1 [39];). We used a mass matching cut-off of ± 1 *m*/*z*, which is conservative given the mass resolution (full width at half maximum) of ~ 2500 that is expected when operating in linear positive mode. We repeated this analysis also for *N. subfulva* to examine whether similar functional distributions were, at least in part, present in the venom gland of this species. For *N. nigricollis*, we also examined the spatial correlation of toxins and their co-correlation with genetic distance. For the genetic distance, we used maximum likelihood pairwise distances calculated during our phylogenetic reconstruction of the identified sequences (see below). We estimated the co-occurrence of 3FTx by calculating the pairwise distance correlates between the peaks corresponding to the identified 3FTx in SCILs Lab. The correlation between genetic distance and spatial correlation was estimated in Prism v9.1.0 (GraphPad) using a two-tailed non-parametric Spearman correlation.

### Phylogenetic analysis

To look for the phylogenetic structure underlying the distribution of 3Ftx in the *N. nigricollis* venom gland, we used IQ-TREE v2.0.6 [40] to reconstruct the molecular phylogeny of the identified 3FTx by maximum likelihood based on the mature toxins aligned using L-INS-i algorithm of MAFFT v7.309 [41] (Additional file 2). The most appropriate evolutionary model was selected using ModelFinder [42], while branch support values were estimated by ultrafast bootstrap using 10,000 replicates [43].

### Venom collection

Venom spits were collected from a single specimen of *N. nigricollis* (Tanzanian origin), *N. pallida* (Kenyan origin), and *H. haemachatus* (captive bred) using a transparent visor from a deconstructed face shield. Spitting was encouraged through a makeshift ‘face’ design, created to maximise the contrast of the eyes at which cobras spit; this was adhered to the back of the visor with clear tape and the resulting set-up was suspended in front of the snake using a snake hook (see Additional file 1: Fig. S2). Snakes were presented with the visor for a maximum of 5 min. Liquid venom droplets collected on the visor were pipetted, and dried venom scraped, into a glass petri dish. The raw weight of the contents was then recorded (‘wet weight’). Immediately following the collection of venom spits, venom was additionally collected by allowing each restrained snake to bite a parafilm-covered glass dish, resulting in expulsion, in a conventional venom collection process known as ‘milking’. The wet weight of the resulting venom was recorded. All venom collection dishes were then sealed with parafilm and stored at − 20 °C overnight, prior to lyophilisation the next day. Subsequently, venom samples were weighed again (‘dry weight’) and stored at 4 °C until use. Lyophilised venoms were reconstituted in phosphate-buffered saline (PBS, pH 7.4) to create a 1-mg/mL working stock for the experiments described below.

### Venom compositional profiling

Comparative compositional profiles of spat and milked venom were generated via the use of (i) cation exchange chromatography, (ii) reversed-phase high-performance liquid chromatography (RP-HPLC), (iii) liquid chromatography-coupled mass spectrometry (LC-MS), and (iv) sodium dodecyl-sulphate polyacrylamide gel electrophoresis (SDS-PAGE). For cation exchange, a preliminary buffer exchange step was performed using a 5-mL Sephadex G25 desalting column (GE Healthcare). This was set up on an AKTA Pure M chromatography system and pre-equilibrated with 0.2 μm filtered 25 mM sodium phosphate. Venom samples were then injected in 500 μL volumes, and the column was run with 50 mM sodium phosphate, pH 6.0 at a flow rate of 0.5 mL/min. Elution was monitored at 280 nm, and 0.5 mL fractions were collected sequentially. From this buffer exchange step, 300 μL of each venom was loaded onto a 1-mL Resource S cation exchange column (GE Healthcare) on an AKTA Pure M chromatography system and equilibrated in 50 mM sodium phosphate, pH 6.0. The proteins were then separated at 0.3 mL/min with a 15-mL gradient of 0–0.75 M NaCl in 50 mM sodium phosphate, pH 6.0. Elution was monitored at 214 nm. For RP-HPLC, a further 85 μL of venoms from the buffer exchange step was made up to 0.05% in trifluoroacetic acid (TFA) and centrifuged at 10,000 × *g* for 5 min. Venom samples were then injected onto a Phenomenex Jupiter C4 RP-HPLC column (250 × 4.6mm) equilibrated in 0.1% TFA. The column was run at 0.7 mL/min, and the proteins were separated over 60 min with a 2–72% gradient of acetonitrile in 0.1% TFA. Elution was monitored at 214 nm.

For the analyses of venoms by LC-MS, lyophilised venoms were dissolved in 0.5% formic acid (FA) at a concentration of 1 mg/mL and analysed by LC-MS using a 5600 TripleTOF (SCIEX, USA) equipped with a Turbo-V source heated to 550 °C. The dissolved samples were fractionated on a Shimadzu (Kyoto, Japan) Nexera UHPLC with an Agilent Zorbax stable-bond C18 column (Agilent, USA) (2.1 × 100 mm, 1.8 μm particle size, 300 Å pore size), using a flow rate of 180 μL/min and a gradient of 1–40% solvent B (90% acetonitrile, 0.1% FA) in 0.1% FA over 15 min, acquiring spectra at *m*/*z* 300–1800 with an accumulation time of 250 ms. Spectra were analysed using PeakView v2.2 (SCIEX).

To analyse venom by SDS-PAGE, we used hand cast 15% tris-polyacrylamide gels. Venom samples were reconstituted to 1 mg/mL with the addition of PBS, reduced by adding equal volume beta-mercaptoethanol-containing sample-loading buffer and incubating for 10 min at 95 °C, before 10 μg venom was loaded. We used 5 μL Broad Range Protein Molecular Marker (10–225 kDa; Promega) as our standard. Gels were run at 110 V, 44 mA for 25 min, then at 200 V for 15 min. The resulting gels were stained with Coomassie blue G250 overnight and destained the following morning.

### Coagulation assay

To quantify the coagulopathic activity of the various venom samples, we used a previously described plasma clotting assay with citrated bovine plasma (Equitech-Bio, SBPUC35-0100) [26]. Plasma was stored at − 80 °C until use and defrosted at 37 °C using a water bath prior to gentle centrifugation. Venom samples were diluted to 100 μg/mL in phosphate-buffered saline (PBS), before the addition of 10 μL in quadruplicate to a Greiner Bio-One clear 384-well microplate. A solution of 20 mM CaCl_2_ was made using deionised water and added (20 μL) to each well using a Thermo Scientific™ Multidrop™ 384 Labsystems multidrop pipette. The multidrop was then flushed with water before being used to overlay the plate with 20 μL of thawed plasma into each well. PBS instead of venom was used as the negative control. The plate was then read kinetically at a wavelength of 595 nm and a temperature of 25 °C using a FLUOstar Omega (BMG Labtech) plate reader, with the run time set to 110 cycles at a cycle time of 95 s. The data was extracted using Multivariate Adaptive Regression Splines® (MARS) software and exported to Microsoft Excel. The anticoagulant activity of each venom was calculated as the area under the curve (AUC) of each sample replicate minus the mean (*m*) control AUC, averaged (*m*(AUC − cAUC)) from assay start to the time taken for plasma to clot under the negative control (101.33 min/65 cycles). A two-way ANOVA followed by Bonferroni corrections was performed in GraphPad Prism 5.0, comparing the milked versus spat venom samples for the tested species, and applying a significance threshold of *P* < 0.05.

### Enzymatic phospholipase assay

To quantify the enzymatic PLA_2_ activity of each venom, a colourimetric absorbance-based assay was used, applying previously described protocols [28]. Briefly, a reaction solution was first prepared using 34 μL of 50 μM Cresol red dye, 26 μL of 0.875 mM Triton X-100, and 1 mL of 5× salt mix (0.29 g NaCl, 0.375 g KCl, 0.55 g CaCl_2_), and then brought to a 5-mL solution using 1 mM Tris base, pH 8.5. Venom samples were diluted in 1 mM 1 mM Tris base, pH 8.5, to a concentration of 10 μg/mL to maintain the pH balance required for the assay. Thereafter, 10 μL of each venom sample was pipetted into wells of a Greiner Bio-One clear 384-well microplate in triplicate, with 10 μL of Tris buffer used as a negative control. Shortly before adding the reaction solution to the plate, 168 μL of the substrate (l-α-phosphatidylcholine; stock concentration 26 mM; SIGMA Life Sciences) was added to the premade reaction solution, and the pH adjusted to 8.5 using either sodium hydroxide or hydrochloric acid, if necessary. Thereafter, 40 μL of this final reaction solution was added to each well on the plate using a Thermo Scientific™ Multidrop™ 384 Labsystems multidrop pipette. Immediately after addition, the plate was read on an FLUOstar Omega plate reader, which was set to a temperature of 25 °C and read kinetically at a wavelength of 572 nm. The number of cycles was set to 65, and the cycle time was 40 s. Following completion, the data was extracted using the MARS software and exported to Microsoft Excel. Enzymatic PLA_2_ activity was measured as the area under the curve (AUC) of each sample replicate minus the mean (*m*) control AUC, averaged (*m*(AUC − cAUC)), from assay start to the time taken for the substrate to completely diminish under the positive control (for which crude Sri Lankan *Daboia russelii* venom was used) (34 min/52 cycles). A two-way ANOVA followed by Bonferroni corrections were performed in GraphPad Prism 5.0, comparing the milked versus spat venoms for the tested species and applying a significance threshold of *P* < 0.05.

To examine the distribution of enzymatic PLA_2_ activity across the *N. nigricollis* venom gland, we followed the protocol for functional MSI (fMSI) recently described by Hamilton et al. [11], using the same venom gland and section preparation as for our conventional MSI analyses. As substrate, we used the glycerophospholipid phosphatidylcholine (PC) 16:0/22:6 (Avanti Polar Lipids, Alabaster, AL, USA). Enzymatic hydrolysis of this PC at the sn-2 position by PLA_2_ produces the lyso-phosphatidylcholine (LPC) 16:0. The substrate was prepared by diluting 50 μL of 100 μM PC in 4 mL 50% methanol and sprayed onto the tissue section for 60 cycles, where each cycle included 2 s spray, 30 s incubation, and 20 s drying time, using an ImagePrep. fMSI data were acquired as per conventional MSI, except that the mass spectrometer was operated in reflectron mode over a mass range of *m*/*z* 160–1400. Data were analysed post-acquisition using Flex Imaging 4.1 and SCILs LAB, using the ratio of LPC16:0 (*m*/*z* 496.6) to PC16:0/22:6 (*m*/*z* 805.5) to indicate PLA_2_ activity.

### Tissue proteomics

To confirm the broad distribution of PLA_2_ across the venom gland, we used the same protocol as described previously for the cobra venom gland [11]. The section was treated with xylene to remove paraffin before it was cut into four equal parts. Tissue was dissolved in 50 mM ammonium bicarbonate 15% acetonitrile (vol/vol) pH 8, cystines reduced by incubation in 5 mM dithiothreitol at 70 °C for 5 min, and alkylated with 10 mM iodoacetamide at 37 °C for 90 min. The reduced and alkylated samples were then digested with 30 μg/μL trypsin overnight at 37 °C, and the resulting tryptic peptides desalted using a C18 ZipTip (Thermo Fisher, USA), dried using vacuum centrifugation, dissolved in 0.5% formic acid (FA), and analysed by liquid chromatography-coupled tandem mass spectrometry (LC-MS/MS) using the same setup as for the LC-MS experiments described above, except that peptides were eluted across a 60-min gradient. MS1 spectra were acquired at 300–1800 *m*/*z* with an accumulation time of 250 ms, while the 20 most intense ions with a charge of + 2 to + 5 and an intensity of at least 120 counts/s were selected for MS2, with a unit mass precursor ion inclusion window of ± 0.7 Da and excluding isotopes within ± 2 Da. MS2 scans were acquired at 80–1400 *m*/*z*, with an accumulation time of 100 ms, and optimised for high resolution. The resulting spectra were searched against all *Naja* sp. sequences from UniProtKB [38] using Protein Pilot 4.5 (SCIEX) set to thorough search but not allowing for amino acid substitutions. We then used the number of high-confidence peptides assigned to venom PLA_2_ as a rough indication of abundance in each tissue section quarter.

### Metalloproteinase assay

The snake venom metalloproteinase (snake venom metalloproteinase (SVMP)) activity of each venom was measured via changes in fluorescence resulting from the cleavage of the target substrate (Mca-K-P-L-G-L-Dpa-A-R-NH_2_ Fluorogenic Peptide Substrate IX; R&D Systems, catalogue number ES010), as previously described [29]. The substrate was reconstituted to a 6.2-mM solution with dimethyl sulfoxide (DMSO), before working concentrations of 10 μM were prepared by diluting 9 μL of 6.2 mM substrate in a 5-mL assay buffer. The assay buffer was prepared from a 50-mL stock of 150 mM NaCl solution containing 50 mM Tris-HCl (pH 7.5–8). The substrate solution was then stored short term in the dark at 4 °C until use. Ten microlitres of each venom sample (100 μg/mL) was added in triplicate to the wells of a Greiner Bio-One clear 384-well microplate, with PBS used as a negative control and crude Brazilian *Bothrops jararaca* venom used as a positive control. Next, 90 μL of the reaction buffer was added to each well using a multi-channel pipette, and a FLUOstar Omega spectrophotometer was used to measure the experimental reaction kinetically at 25 °C, with an excitation wavelength of 320 nm and emission wavelength of 405 nm. The gain adjustment was set to 850 prior to measurement, and the assay was run for 160 cycles at 22 s per cycle. After the plate was read, the data was extracted using the MARS software and exported to Microsoft Excel. The SVMP activity was measured as the area under the curve (AUC) of each sample replicate minus the mean (*m*) control AUC, averaged (*m*(AUC − cAUC)), from assay start to the time taken for the substrate to completely diminish under the positive control (30 min/82 cycles). A two-way ANOVA followed by Bonferroni corrections was performed in GraphPad Prism 5.0, comparing the milked versus spat venoms for the tested species, and applying a significance threshold of *P* < 0.05.

### Cell viability assays

Immortalised human keratinocytes (HaCaT cells) were seeded at 20,000 cells/well in 96-well plates on day 1 and left to adhere in a humidified incubator overnight at 37 °C and 5% CO_2_ (standard conditions). On day 2, the cells were treated in triplicate with various concentrations of the spat or milked venom samples (5.63–75.00 μg/mL; diluted from 10 mg/mL stocks into 10% foetal bovine serum (FBS), 1% penicillin/streptomycin, 2% sodium pyruvate containing DMEM cell growth medium, referred to hereon as ‘Standard Medium’) and then left in the incubator for 24 h at standard conditions. A no venom-vehicle (PBS) treatment in Standard Medium was used as the positive control, and a PBS-only treatment was used as the negative control. On day 3, MTT (3-(4,5-dimethylthiazol-2-yl)-2,5-diphenyl tetrazolium bromide) cell viability assays [27] were performed. Briefly, a 5-mg/mL MTT solution in PBS was prepared and filtered to remove impurities, and then diluted to 0.83 mg/mL in Standard Medium (MTT-containing Standard Medium). After the 24-h venom treatments were completed, the venom-containing Standard Medium was aspirated from all wells, the wells were rinsed with 100 μL of PBS, the PBS removed and then 120 μL of MTT-containing Standard Medium added to each well. The plates were placed back in the incubator for 30 min to allow the MTT cell metabolism reaction to occur, after which the MTT-containing Standard Medium was removed from each well and replaced with 100 μL of DMSO. The plates were then shaken and the absorbance at 550 nm (A_550_) quantified using a FLUOstar Omega Microplate Reader (BMG Labtech). The %-cell viability at each venom concentration was calculated by dividing the resulting triplicate-averaged A_550_ reading by that of the Standard Medium-treated controls, multiplied by 100%. The concentration that resulted in a 50% reduction in viability (IC_50_) was calculated from the concentration versus response curves using the ‘[Inhibitor] vs normalised response – variable slope’ function in GraphPad Prism, which uses the following equation:

$$Y=100/\left(1+{\left(\frac{{\mathrm{IC}}_{50}}{X}\right)}^{\mathrm{HillSlope}}\right)$$

where *X* is the concentration of venom, *Y* is the normalised %-cell viability (with a 100% maximum and 0% minimum) decreasing as *X* increases, and HillSlope is the GraphPad-calculated slope factor. Three separate trials were completed for each venom, and within each trial, individual treatments were also performed in triplicate. A two-way ANOVA followed by Bonferroni corrections was performed in GraphPad Prism 5.0, comparing the extracted versus spat venoms for the tested species, and applying a significance threshold of *P* < 0.05.

## Supplementary Information

**Additional file 1: Fig. S1.** Normalised across-tissue averaged spectrum across the full acquired range of m/z. **Fig. S2.** The experimental setup for collecting spat venom. **Fig. S3.** A summary of the weight of spat and milked venom collected from the spitting cobras used in this study. **Fig. S4.** Profiles of spat and milked venoms measured via cation exchange chromatography and RP-HPLC. **Fig. S5.** Coagulation profiles of spitting cobra venoms on citrated bovine plasma. **Fig. S6.** Cell viability assays. **Fig. S7.** Enzymatic phospholipase assay absorbance curves. **Fig. S8.** Snake venom metalloproteinase assay fluorescence curves. **Fig. S9.** PLA_2_ is evenly distributed through the length of the venom gland of *N. nigricollis*. **Table S1.** Three finger toxins identified by mass spectrometry imaging. **Table S2.** Spatial correlation and genetic distances of three finger toxins identified by mass spectrometry imaging of *N. nigricollis* venom gland.

**Additional file 2.** Multiple sequence alignment of identified mature 3FTx used for phylogenetic and genetic distance analyses. Toxins are named according to observed m/z, with the names corresponding to those listed in Additional file 1: Table S1.

**Additional file 3.** Raw absorbance data and area under the curve (AUC) values for the plasma coagulation assay, corresponding to data presented in Fig. 2D and Additional file 1: Fig. S5.

**Additional file 4.** Raw % cell viability and IC50 values for the MTT viability assay, corresponding to data presented in Fig. 2E and Additional file 1: Fig. S6.

**Additional file 5.** Raw absorbance data Area Under the Curve (AUC) values for the enzymatic PLA_2_ assay, corresponding to data presented in Fig. 2F and Additional file 1: Fig. S7.

**Additional file 6.** Raw fluorescence values for the SVMP assay, corresponding to Additional file 1: Fig. S8.

## Data Availability

All data generated or analysed during this study are included in this published article, its supplementary information files, and publicly available repositories. All genetic data used herein were obtained from public databases, and information on *N. nigricollis* and *N. subfulva* used in this study is available under NCBI BioProject accession number PRJNA506018 [39]. The LC-MS, LC-MS/MS, proteomic, MSI, and fMSI data have been deposited to the ProteomeXchange Consortium via the PRIDE [44] partner repository with the dataset identifier PXD034174. Data points for the raw absorbance data and area under the curve (AUC) values for the plasma coagulation assay in Fig. 2D and Additional file 1: Fig. S5 are provided in Additional file 3. Data points for the raw % cell viability and IC_50_ values for the MTT viability assay in Fig. 2E and Additional file 1: Fig. S6 are provided in Additional file 4. Data points for the raw absorbance data area under the curve (AUC) values for the enzymatic PLA_2_ assay in Fig. 2F and Additional file 1: Fig. S7 are provided in Additional file 5. Data points for the raw fluorescence values for the SVMP assay in Additional file 1: Fig. S8 are provided in Additional file 5. Further information and requests for resources and reagents should be directed to and will be fulfilled by the lead contact, Eivind A. B. Undheim (e.a.b.undheim@ibv.uio.no).

## Abbreviations

3FTx: Three-finger toxins
ANOVA: Analysis of variance
AUC: Area under the curve
CHCA: α-Cyano-4-hydroxycinnamic acid
DMSO: Dimethyl sulfoxide
FA: Formic acid
FBS: Foetal bovine serum
fMSI: Functional mass spectrometry imaging
H&E: Haematoxylin and eosin
ITO: Indium-tin oxide
LC-MS: Liquid chromatography-coupled mass spectrometry
LC-MS/MS: Liquid chromatography-coupled tandem mass spectrometry
LPC: Lysophosphatidylcholine
m/z: Mass to charge ratio
MALDI: Matrix-assisted laser desorption ionisation
MARS: Multivariate Adaptive Regression Splines®
ML: Maximum likelihood
MSI: Mass spectrometry imaging
MTT: Methyl tetrazolium
PC: Phosphatidylcholine
PBS: Phosphate-buffered saline
PLA_2_: Phospholipase A2
pLSA: Probabilistic latent semantic analysis
RP-HPLC: Reversed-phase high-performance liquid chromatography
SDS-PAGE: Sodium dodecyl-sulphate polyacrylamide gel electrophoresis
SEM: Standard error of the mean
SVMP: Snake venom metalloproteinase
TFA: Trifluoroacetic acid
TICs: Total ion counts
