# Supplementary material for: Physiological constraints dictate toxin spatial heterogeneity in snake venom glands
Source: BMC Biol. 2022 Jun 27;20:148. doi: 10.1186/s12915-022-01350-y (PMC9238143; doi:10.1186/s12915-022-01350-y)
Supplement: Supplementary file 1 — Additional file 1: Fig. S1. Normalised across-tissue averaged spectrum across the full acquired range of m/z. Fig. S2. The experimental setup for collecting spat venom. Fig. S3. A summary of the weight of spat and milked venom collected from the spitting cobras used in this study. Fig. S4. Profiles of spat and milked venoms measured via cation exchange chromatography and RP-HPLC. Fig. S5. Coagulation profiles of spitting cobra venoms on citrated bovine plasma. Fig. S6. Cell viability assays. Fig. S7. Enzymatic phospholipase assay absorbance curves. Fig. S8. Snake venom metalloproteinase assay fluorescence curves. Fig. S9. PLA2 is evenly distributed through the length of the venom gland of N. nigricollis. Table S1. Three finger toxins identified by mass spectrometry imaging. Table S2. Spatial correlation and genetic distances of three finger toxins identified by mass spectrometry imaging of N. nigricollis venom gland. [file 12915_2022_1350_MOESM1_ESM.pdf]

Supplemental Information for:

Physiological constraints dictate toxin spatial heterogeneity in snake venom glands

Taline D. Kazandjian1, Brett R. Hamilton2,3, Samuel D. Robinson2,4, Steven R. Hall1, Keirah E. Bartlett1, Paul Rowley1, Mark C. Wilkinson1, Nicholas R. Casewell1, Eivind A. B. Undheim2,4,5,6,*

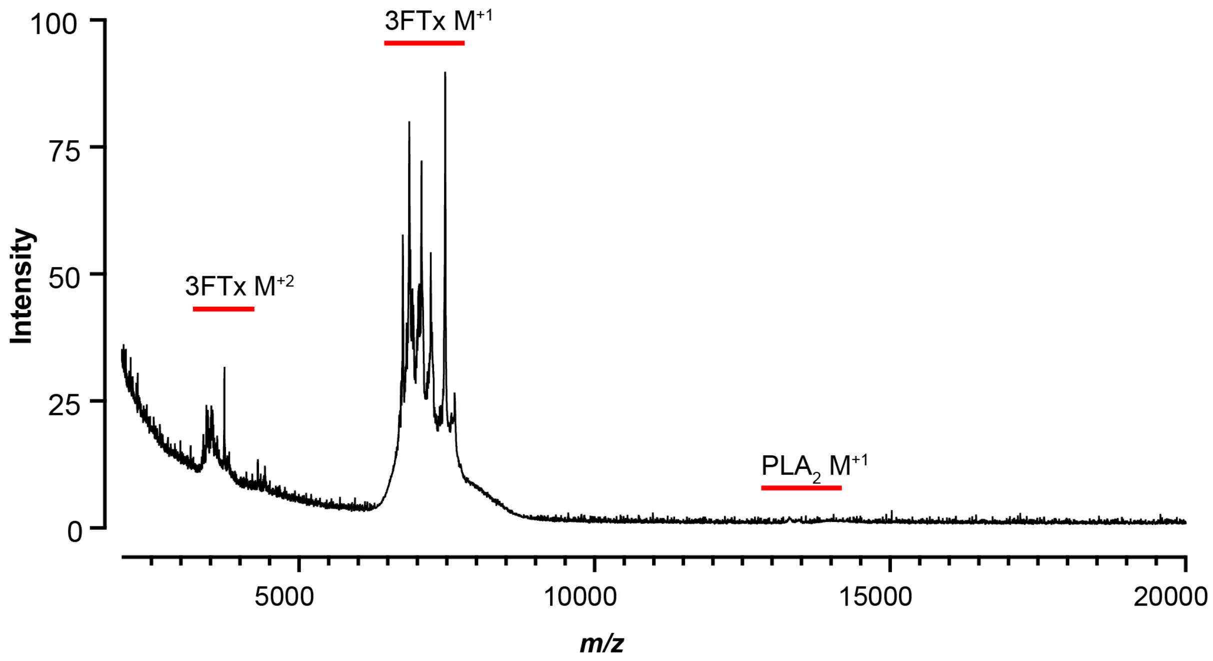

Fig. S1. Normalised across-tissue averaged spectrum across the full acquired range of m/z. Spectrum is from the same acquisition as is shown in Fig. 1. Mass ranges of major venom components are displayed above the spectrum. M^{+2} indicates doubly charged.

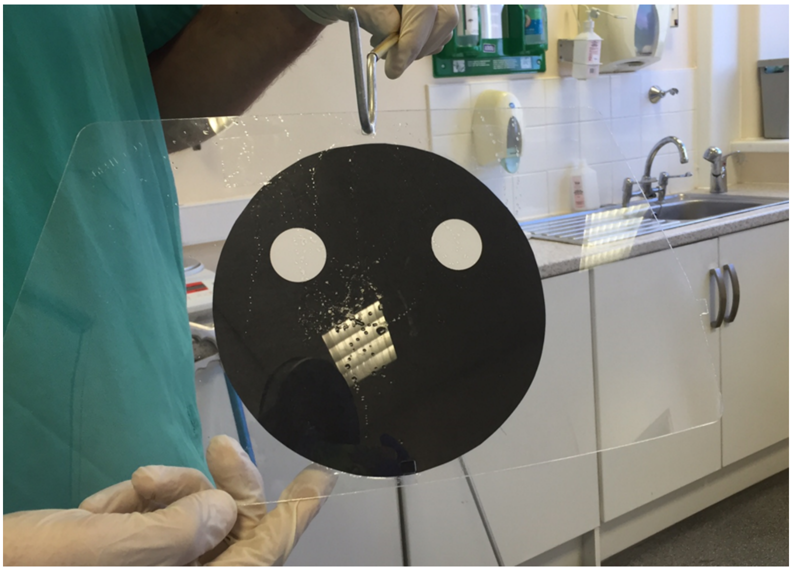

Fig. S2. The experimental setup for collecting spat venom. Spitting cobras readily spit at human faces, so to collect venom spits, a sterile isolated mask visor with paper bearing a crude "face" design was hung from a snake hook. Ejected venom was collected from the resulting visor. The image shows the visor and spat venom.

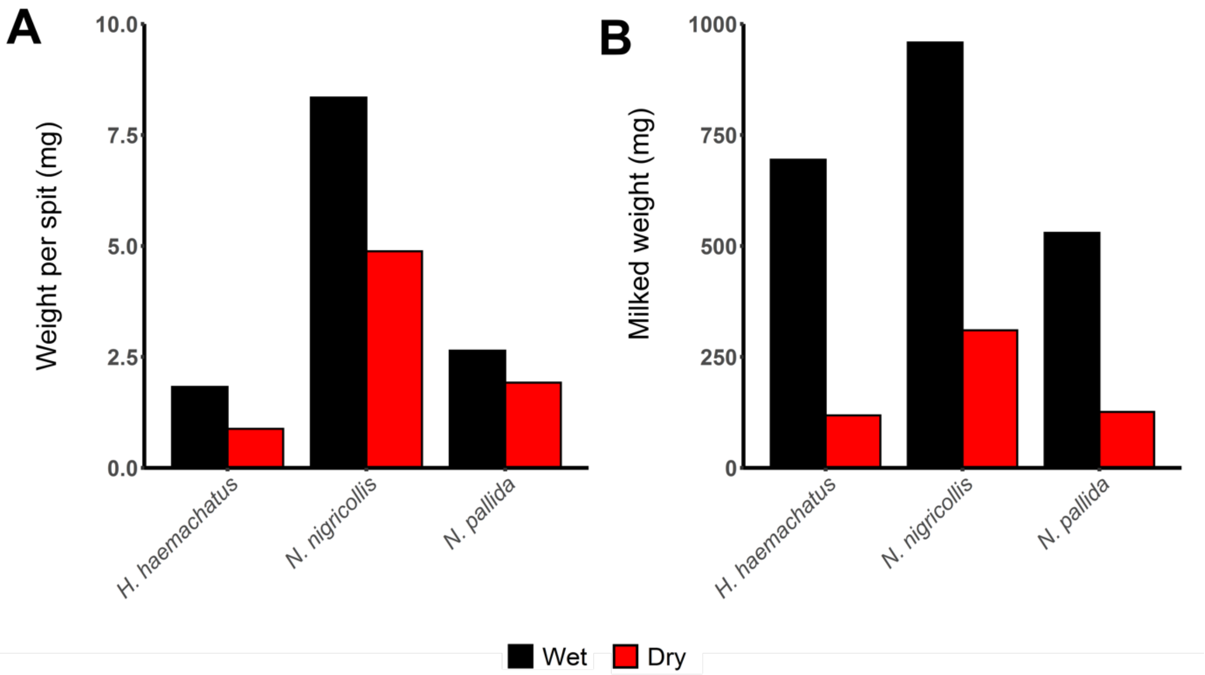

Fig. S3. A summary of the weight of A) spat and B) milked venom collected from the spitting cobras used in this study. Spat venom was collected by stimulating individual snakes to spit at a visor mask. Due to large variation in the number of spits collected per individual (between 2 and 16 spits, measured across 2 encounters), spat venom is represented here as mean weight per spit. Milked venom was collected by inducing the snakes to bite on to a parafilm covered glass dish immediately after the collection of venom spits.

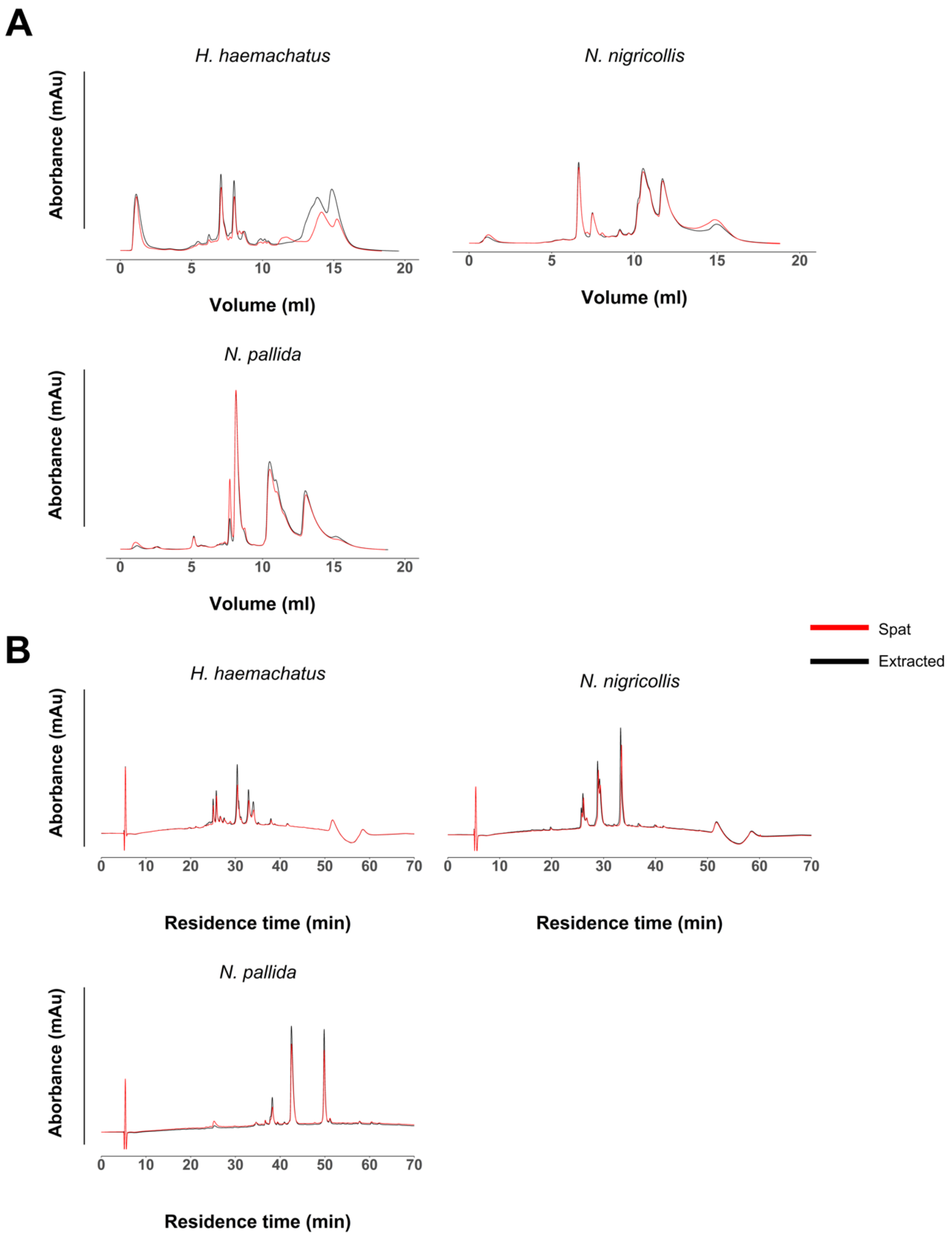

Fig. S4. Profiles of spat and milked venoms measured via A) cation exchange chromatography B) RP-HPLC. For the cation exchange step (A), 300 µL desalted venom samples were loaded onto a 1 mL Resource S cation exchange column and equilibrated in 50 mM sodium phosphate, pH 6.0. The proteins were separated at 0.3 mL/min with a 15 mL gradient of 0-0.75 M NaCl in 50 mM sodium phosphate, pH 6.0 and elution monitored at 214 nm. For RP-HPLC (B), 85 µL desalted venom samples were injected onto a Phenomenex Jupiter C4 RP-HPLC column (250 x 4.6mm) equilibrated in 0.1% TFA. The column was run at 0.7 mL/min and the proteins were separated over 60 minutes with a 2–72% gradient of acetonitrile in 0.1% TFA. Elution was monitored at 214 nm.

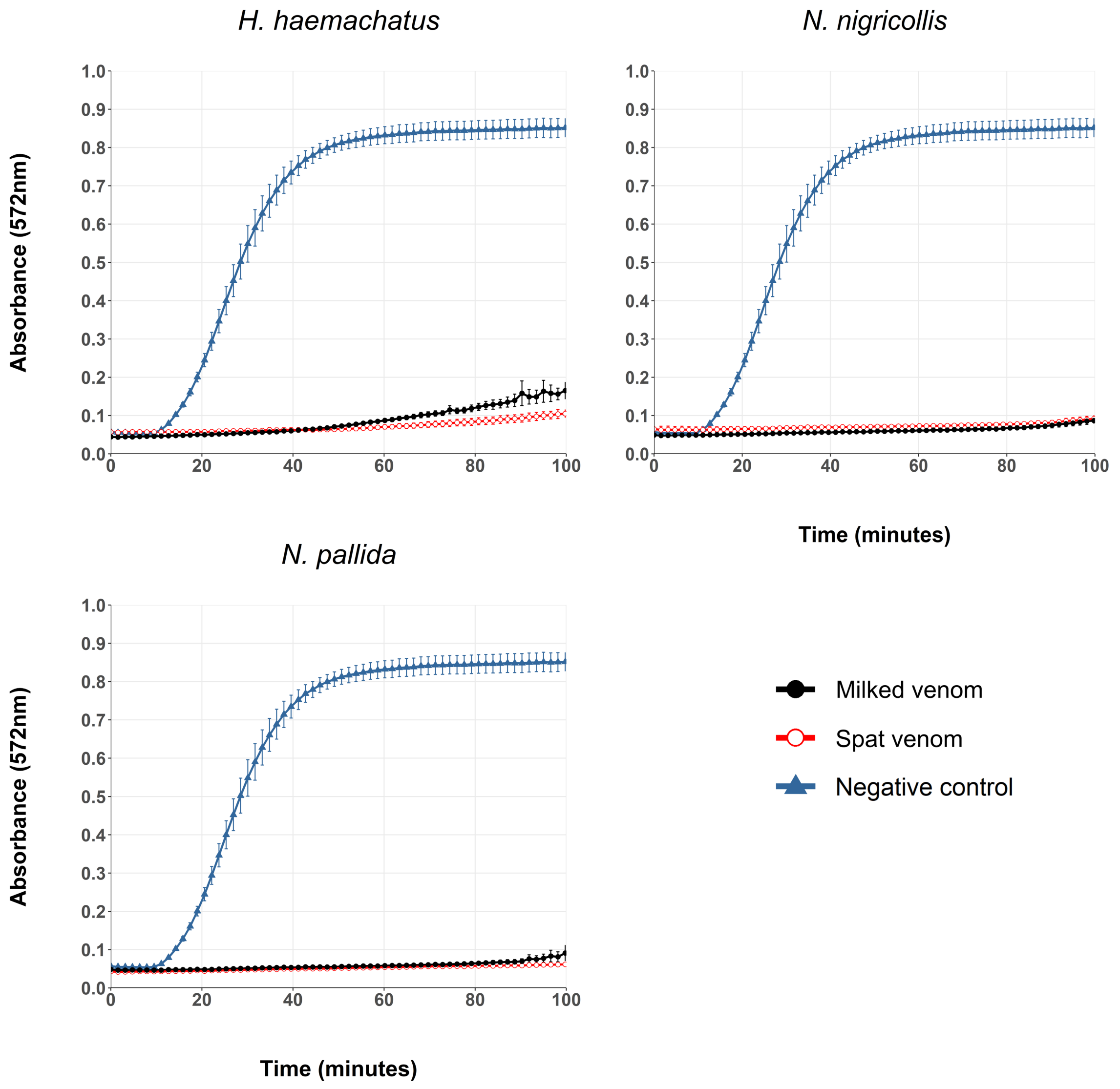

Fig. S5. Coagulation profiles of spitting cobra venoms on citrated bovine plasma. Data points represent light absorbance through plasma at a wavelength of 595 nm. Each sample well contained 1 µg of spat or milked venom in replicates of four (see Additional file 3). Negative control wells contained phosphate-buffered saline (PBS). Error bars show standard error of the mean.

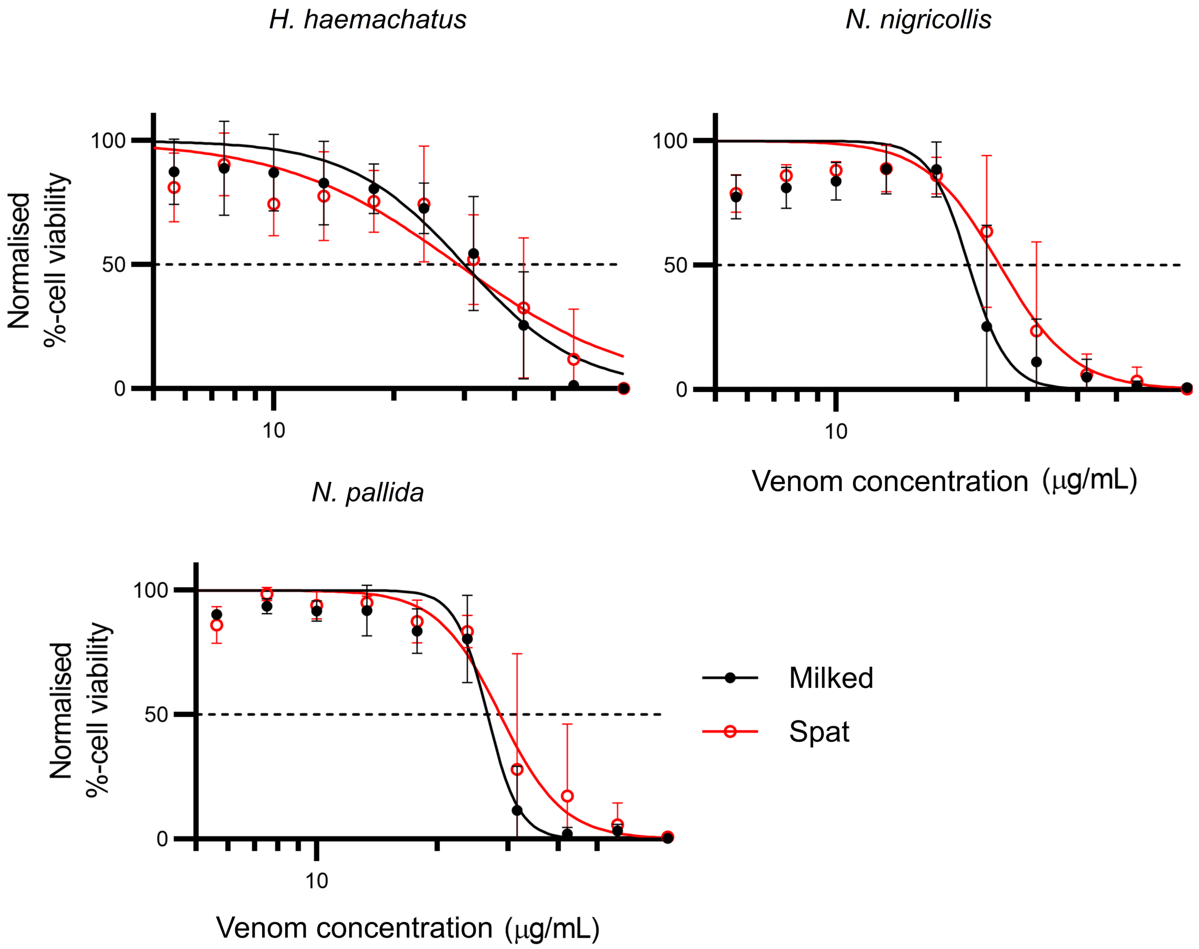

Fig. S6. Cell viability assays. Assays were performed on immortalised human keratinocytes, applying spat or milked venom samples in triplicate in a concentration gradient from 5.63 μg/mL to 75 μg/mL. Phosphate-buffered saline (PBS) was used as a negative control. Error bars show standard error of the mean. For individual datapoints see Additional file 4.

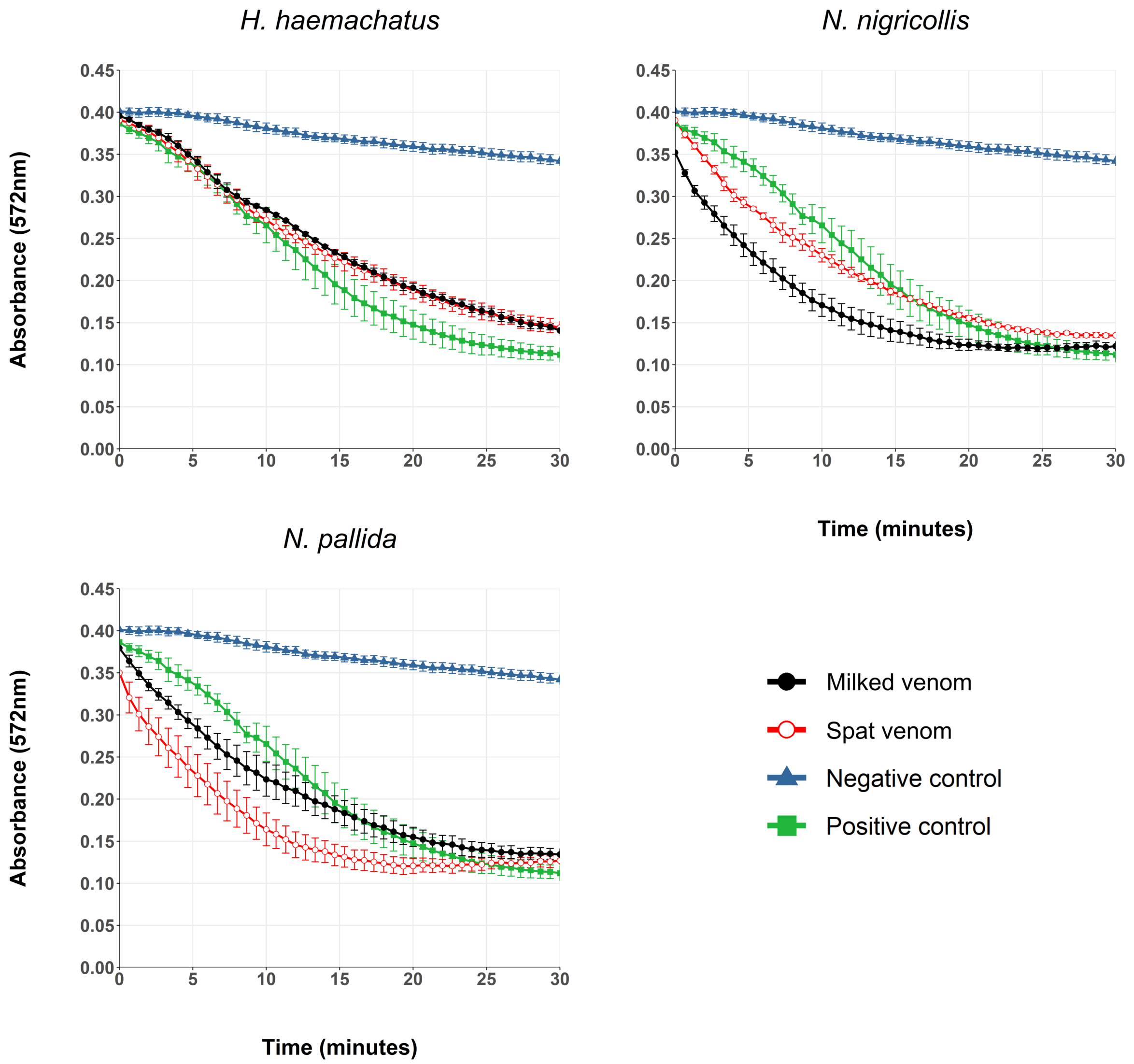

Fig. S7. Enzymatic phospholipase assay absorbance curves. Data points represent concentration of artificial substrate (L-α-phosphatidylcholine) measured as light absorbance at 572 nm. Each well contained 1µg spat or milked venom in triplicate. Phosphate-buffered saline (PBS) was used as a negative control. Error bars show standard error of the mean. For individual datapoints see Additional file 5.

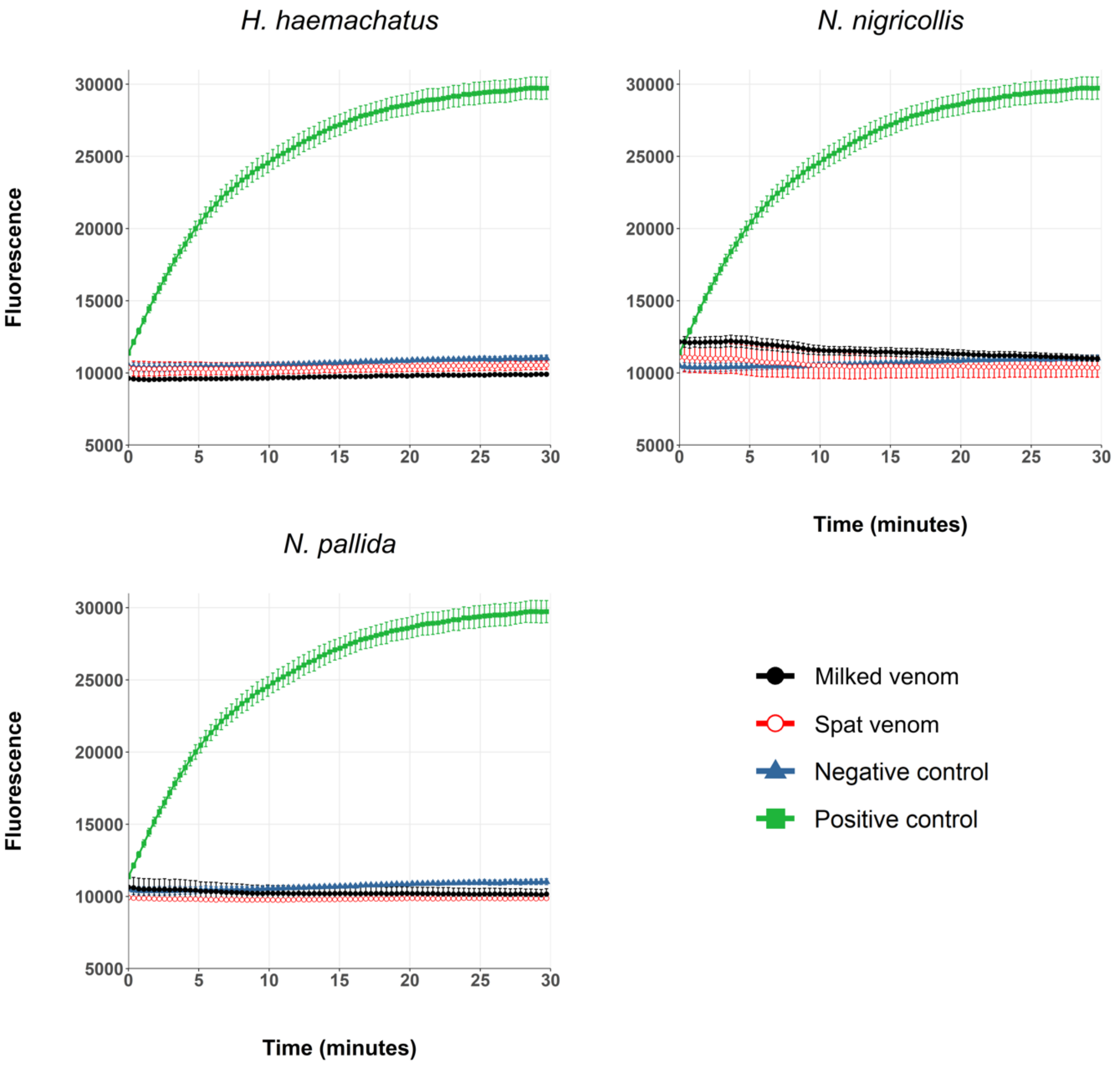

Fig. S8. Snake venom metalloproteinase assay fluorescence curves. Data points represent concentration of cleaved artificial fluorogenic substrate measured as light absorbance through fluorescence measured at an excitation wavelength of 320 nm and emission wavelength of 405 nm. Each well contained 1µg spat or milked venom in triplicate. Phosphate-buffered saline (PBS) was used as a negative control. Error bars show standard error of the mean. For individual datapoints see Additional file 6.

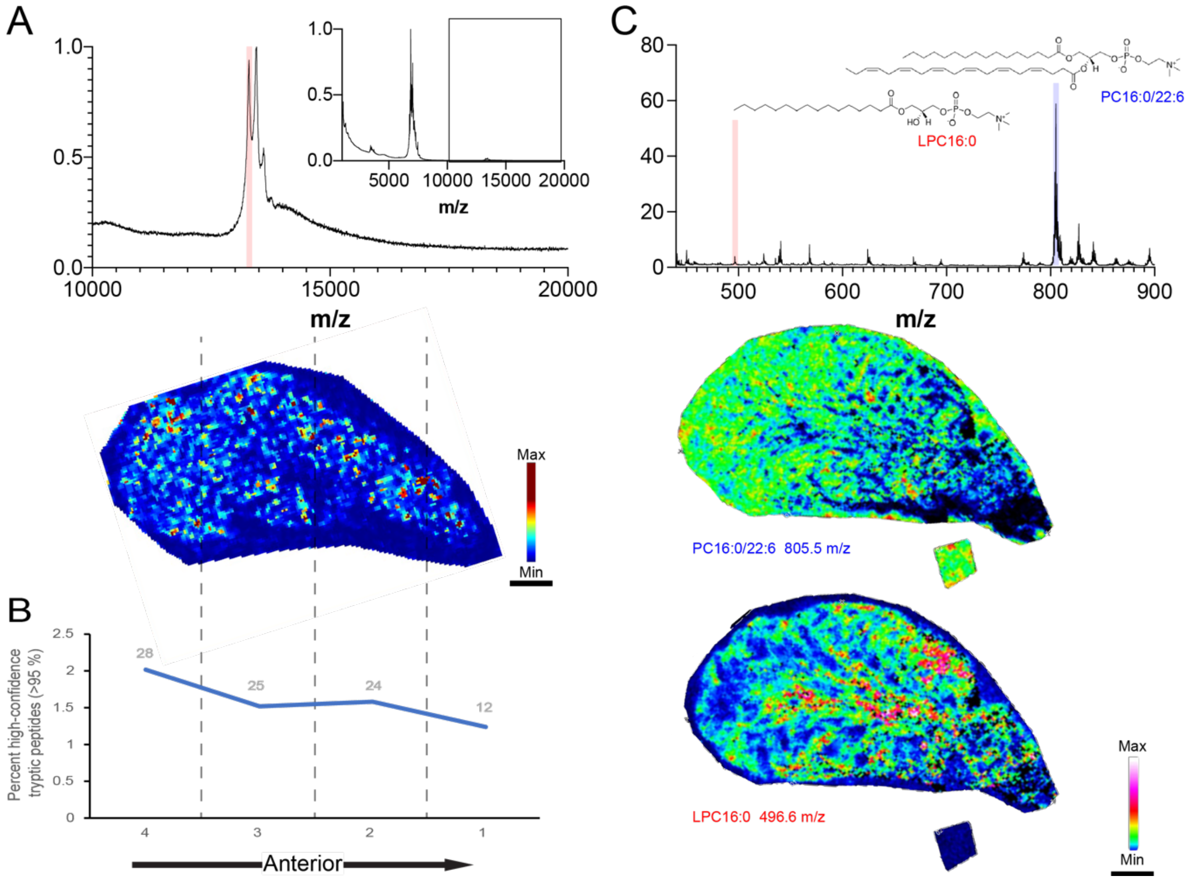

Fig. S9. PLA$_2$ is evenly distributed through the length of the venom gland of ***N. nigricollis*****.** A) Longitudinal section of the same venom gland as in Fig. 1 showing the distribution of 13293 m/z, which is a likely isoform of *N. nigricollis* venom basic PLA$_2$ (UniProtKB accession P00605). The distribution is shown as a heatmap of normalised intensities according to the colour gradient legend. The corresponding peak is highlighted in the across-tissue averaged spectrum above, zoomed in from the full acquired spectrum (boxed, inset). B) The distribution of the peaks corresponding to venom PLA$_2$ is in agreement with analyses by shotgun-proteomics of parts of adjacent sections from the same gland and the roughly approximated abundances as indicated by the percentage of total high-confidence matches accounted for by hits to venom PLA$_2$ paralogues. The parts of the gland used for separate proteomic analyses are indicated with dashed lines and are aligned with the graphed proportions of high-confidence hits as well as the section used for MSI. Absolute number of high-confidence peptides are given at each point. C) This distribution is further corroborated by the distribution of PLA$_2$ activity throughout the length of the gland. The distributions of the glycerophospholipid phosphatidylcholine (PC) 16:0/22:6 applied as PLA$_2$ substrate (m/z 805.5; middle) and the lyso-phosphatidylcholine (LPC) 16:0 (m/z 496.6; bottom) resulting from hydrolysis of by PLA$_2$ are shown as heatmaps of normalised intensities according to the colour gradient legend. Tissue-wide average spectrum is shown at the top while the off-tissue controls for both extracted masses is shown below each section image.

Table S1 Three finger toxins identified by mass spectrometry imaging. All accession numbers refer to contig names of sequences obtained from bioproject PRJA506018, except the UniProtKB accessions P0DSN1 and P01452. Predicted (Pred) and observed (Obs) m/z correspond to singly charged ions (M+1).

Name	Accession	Pred m/z	Obs m/z	Mature toxin sequence
Putative cytotoxin	T3430	6972.19	**6971**	RQCTQQKPPFYMNCPEGMNVCYTIIFSPFKFYTKRGCAATCPKSRVRAKIECCEKDRCNS
Cytotoxin 6-like	T2771	6688.11	**6688**	LKCNQLIPPFFKTCPEGKNLCYKMTMRVGPRVPVKRGCASTCPKSNALMKVVCCNTDRCN
Cytotoxin 1	T1107	6820.41	**6820**	LKCNQLIPPFWKTCPKGKNLCYKMTMRAAPMVPVKRGCIDVCPKSSLLIKYMCCNTDKCN
Naniproin	P0DSN1, T1821	6888.47	**6888**	LKCNRLIPPFWKTCPEGKNLCYKMTMRLAPKVPVKRGCIDVCPKSSLLIKYMCCTNDKCN
Cytotoxin 4b	T1148	6756.32	**6756**	LKCNKLIPIAYKTCPEGKNLCYKMMMVSKKMVPVKRGCIDVCPKDSALVKYVCCSTDRCN
Cytotoxin 4	P01452 [+ 2 Da]	6709.25	**6710**	LKCNKLIPIAYKTCPEGKDLCYKMMLASKKMVPVKRGCINVCPKNSALVKYVCCSTDRCN
Cytotoxin 5	T3171	6816.35	**6816**	LKCKKLIPLFSKTCPEGKNLCYKMMIGSKKMVPVKRGCIDVCPKSSFLVKYECCDTDRCN
Short neurotoxin 1	T0696	7069.83	**7070**	LDCHNQQSSEPPTTTRCSSGETNCYKKRWRDHRGYRTERGCGCPTVKKGIQLHCCTTDNCNN
Short neurotoxin 3	T0206	7211.16	**7211**	LNCHNQMSAQPPTTTRCSRWETNCYKKRWRDHRGYKTERGCGCPTVKKGIQLHCCTSDNCNN
Putative muscarinic toxin 2	T0298	7792.86	**7793**	TKCYNHLSRTPETTEICPYSWHFCYKMSWVDGHEGRIERGCTFTCPELRPNGKYVYCCRRDKCNQ
Putative neurotoxin 3	T0074	7368.6	**7369**	RLCLSDYSIFSETIEICPDGHNFCFKKFPKGITRLPWVVRGCAATCPKAEAQVYVDCCARDKCNR
Oxiana-like weak toxin	T0797	7259.53	**7259**	CLNCPEKYCNKVHTCRKGENICFKKFDQRKLLGKRYTRGCAATCPVAKPREIVECCSTDRCNH
Putative neurotoxin 2	T2761	7454.74	**7455**	LTCLICPEKYCNKIHTCRNGENQCFKRFYEGNLLGKRYIRGCAATCPVAKPREIVECCSTDKCNR
Unknown activity	T1809	7072.12	**7072**	LRCYSCGRNGCRDIVTCSEKQEFCFNRFGKRIQRRTSGCAVKCNPRDSLNAHIQCCTTDLCN
Unknown activity	T0799	7283.55	**7284**	MECYKCGASGCHLKITCSAEEKFCYKWKNKISKLRWHGCAKTCTEEDSWKAYIKCCTTNLCNI
Putative muscarinic toxin 1	T0824	7342.5	**7342**	LTCVKEKSIFSDTMEICSDGQNLCFKRWHMVVPGRYKKTRGCAATCPIAENRDVIECCSTDKCNN

Table S2. Spatial correlation and genetic distances of three finger toxins identified by mass spectrometry imaging of *N. nigricollis* venom gland. Toxins are named according to observed m/z, with the names corresponding to those listed in Table S1. Spatial correlations are given above the diagonal, estimated from pairwise correlations of each observed m/z ± 2.267 Da. Pairwise ML distances are given below the diagonal.

	**6688**	**6710**	**6756**	**6816**	**6820**	**6888**	**6972**	**7070**	**7072**	**7211**	**7259**	**7284**	**7342**	**7369**	**7455**	**7793**
**6688**		0.0171	0.411	0.38	0.35	0.119	0.0425	0.0327	0.0219	0.114	0.137	0.0134	0.149	0.0818	0.0472	0.0484
**6710**	0.76		0.00327	0.0222	0.0683	0.226	0.03	0.115	0.0912	0.135	0.00411	0.0859	0.0266	0.00442	0.00588	0.0128
**6756**	0.74	0.09		0.832	0.76	0.392	0.292	0.00137	3.83E-05	0.0883	0.177	0.0097	0.279	0.214	0.0812	0.0962
**6816**	0.77	0.32	0.28		0.929	0.583	0.352	0.000314	0.00119	0.0552	0.17	0.0251	0.217	0.206	0.106	0.0648
**6820**	0.42	0.63	0.55	0.42		0.697	0.398	0.00937	0.0129	0.0201	0.136	0.0381	0.18	0.189	0.114	0.0466
**6888**	0.50	0.69	0.61	0.50	0.14		0.492	0.0919	0.083	0.0188	0.0298	0.0454	0.0445	0.0851	0.0352	0.0256
**6972**	1.93	2.41	2.53	1.93	2.47	2.57		0.122	0.116	0.056	0.00155	0.00415	0.0188	0.0461	0.000115	0.0112
**7070**	4.17	4.77	4.81	4.61	4.41	4.43	4.71		0.949	0.716	0.353	0.0225	0.0306	0.133	0.107	0.0967
**7072**	5.17	5.11	5.05	5.32	5.62	5.05	5.21	5.06		0.674	0.353	0.0281	0.0189	0.118	0.135	0.103
**7211**	4.83	5.54	5.58	5.41	4.91	4.78	5.10	0.18	5.97		0.458	0.0109	0.126	0.25	0.00758	0.139
**7259**	4.01	4.72	4.53	4.57	5.12	6.29	4.07	5.27	3.06	6.12		0.171	0.136	0.311	0.000467	0.106
**7284**	4.95	6.99	6.82	6.05	6.43	6.20	5.97	5.16	2.27	6.81	5.32		0.0072	0.0537	0.000354	0.00422
**7342**	2.92	4.40	4.14	4.37	4.15	4.18	4.38	3.58	3.98	3.96	2.23	5.32		0.14	0.0602	0.0612
**7369**	2.85	3.69	3.69	4.13	4.26	3.96	3.50	5.04	3.92	5.45	3.02	6.72	1.74		0.000327	0.0917
**7455**	4.21	5.01	4.97	4.88	5.23	6.35	4.60	5.30	3.77	6.50	0.29	5.75	2.06	2.91		0.0254
**7793**	3.76	4.77	4.66	5.24	4.92	4.87	5.11	2.99	7.02	3.13	5.34	7.67	4.20	3.26	5.80	
